# Bleomycin-loaded folic acid-conjugated nanoliposomes: a novel formulation for targeted treatment of oral cancer

**DOI:** 10.3389/fbioe.2025.1535793

**Published:** 2025-04-14

**Authors:** Elham Saberian, Janka Jenčová, Andrej Jenča, Andrej Jenča, Fateme Salehipoor, Hadi Zare-Zardini, Adriána Petrášová, Peter Džupa, Meysam Ebrahimifar, Mohammadreza Allahyartorkaman, Jozef Jenča

**Affiliations:** ^1^ Pavol Jozef Šafárik University, Clinic of Dentistry and Maxillofacial Surgery Academy of Košice, Kosice, Slovakia; ^2^ Department of Medicine, Najafabad Branch, Islamic Azad University, Najafabad, Iran; ^3^ Department of Biomedical Engineering, Meybod University, Meybod, Iran; ^4^ Department of Medicine, Slovak Medical University in Bratislava, Bratislava, Slovakia; ^5^ Department of Toxicology, Faculty of Pharmacy, Islamic Azad University, Shahreza Branch, Shahreza, Iran; ^6^ Department of Life Science, College of Life Science, National Taiwan University, Taipei, Taiwan

**Keywords:** nanoliposomes, folate receptor, targeted drug delivery, bleomycin, cancer therapy

## Abstract

**Introduction:**

Targeted delivery of anticancer drugs holds great promise for enhancing therapeutic efficacy while minimizing adverse effects. The folate receptor (FR)-mediated approach offers a selective strategy to target cancer cells overexpressing FR. Bleomycin, an established antitumor antibiotic, suffers from limited efficacy due to poor diffusion into tumor cells. This study examined the anti-cancer potential of folate-targeted liposomal Bleomycin (FL-BLEOMYCIN) in comparison to non-targeted L-BLEOMYCIN on oral cavity cancer (CAL27). The study also investigated FL-Bleomycin’s capacity to halt the cell cycle in the G2/M phase using flow cytometry.

**Methods:**

FL-Bleomycin was produced using thin-layer hydration, followed by incorporation of folic acid into nanoliposomes. To evaluate the release profile, drug release tests were carried out. Cytotoxicity of FL-Bleomycin, L-Bleomycin, and traditional Bleomycin was evaluated using cell viability assays. The cell cycle arrest caused by FL-Bleomycin was examined using flow cytometry. Finally, FL-Bleomycin uptake studies were performed to assess the internalization of FL-Bleomycin by CAL27 cells.

**Results:**

Compared to L-Bleomycin and traditional Bleomycin, FL-Bleomycin showed noticeably more cytotoxicity against CAL 27 cells. The effective arrest of CAL 27 cells in the G2/M phase of the cell cycle by FL-Bleomycin was verified by flow cytometry. Uptake studies revealed increased internalization of FL-Bleomycin by CAL 27 cells compared to standard formulations. Drug release studies showed a consistent, non-explosive release profile. Cells treated with these nanoliposomes, compared to control groups, exhibited a dose-dependent decrease in the intensity of the 170-kDa EGF-R band as observed by Western blot analysis.

**Discussion:**

The findings suggest that FL-Bleomycin is a potential method for delivering drugs precisely in tumors expressing folic acid receptors. Its potential for successful cancer treatment is shown by its higher internalization, improved cytotoxicity, and cell cycle prevention in CAL 27 cells. To find out how effective FL-Bleomycin is *in vivo* and whether it may be used to treat other FR-expressing tumors, more research is necessary.

## Introduction

A common anticancer drug used to treat different kinds of cancer is Bleomycin (Moeller et al., 2008). Its cytotoxic mechanism is the induction of DNA cleavage, which mainly affects cells in the G2 phase of mitosis. Bleomycin has a concise half-life in the bloodstream, which is less than 4 h (He et al., 2023; Chiani et al., 2018). Due to the limited permeability of cell membranes to Bleomycin, frequent injections are usually required for maximum therapeutic efficacy. However, administering higher doses of the drug may increase the likelihood of adverse effects (Tozon et al., 2005). Active targeting, the targeted delivery of drugs, has attracted considerable interest because of its pivotal significance in improving the therapeutic effect of anticancer drugs (Saberian et al., 2024; Haghi Karamallah et al., 2024; Ghanbarikondori et al., 2024). Several efforts have been made to develop innovative Bleomycin delivery systems with the aim of improving its efficacy and reducing toxicity. These initiatives include the development of fusogenic DOPE liposomes to enhance Bleomycin's effectiveness in a mouse model of breast cancer, Liposomal Bleomycin formulation, the development of synthetic liposomes for targeted distribution to the respiratory tract to lessen lung toxicity in mice, the investigation of Bleomycin’s pharmacokinetics and biodistribution in male Balb/c mice, as well as treatments for Lewis lung cancer, using chitosan-based polymeric vesicles (Koshkaryev et al., 2013; Gwinn et al., 2011; Chiani et al., 2017a). The creation of novel Bleomycin formulations to address contemporary issues has drawn more attention in recent years. However, many of these formulations have problems such as poor loading or encapsulation, reduced potency, stability issues and poor retention. Several researchers have investigated the promise of liposomal Bleomycin to address these challenges (Baglo et al., 2014; Kavaz et al., 2010). Liposomal Bleomycin was found by Arndt et al. to be much more effective against Lewis lung cancer and P388 leukemia. However, in the treatment of B16 melanoma, the efficacy was almost identical to that of unencapsulated Bleomycin (Arndt et al., 2001). Alomrani et al. investigated how liposomal composition affected the cytotoxic efficacy of Bleomycin against the Burkitt’s lymphoma cell line Daudi, they discovered that different liposome compositions were differentially effective; fusogenic, flexible or pegylated vesicles showed higher efficacy (Alomrani et al., 2011). There is great interest in drug delivery systems (DDS) that precisely distribute drugs at specific doses and intervals. Among these systems, lipid nanoparticles, including liposomes, have demonstrated exceptional efficacy as drug carriers in therapeutic settings, several of which have been approved by the FDA (Karamallah et al., 2021; Alemi et al., 2018). When it comes to precise medicine delivery, the folate receptor (FR), a membrane-bound protein that is typically not present in normal cells and frequently overexpressed in various epithelial malignancies, is used as an important tumor marker. To target FR-positive tumor cells, folic acid has been conjugated with a variety of drugs, imaging agents and drug carriers. Several formulations, such as farletuzumab (MORAb-003), EC20 and EC145, have reached phase III clinical trials (Peer et al., 2020). This study’s objective was to create a unique nanoliposomal formulation of Bleomycin targeting the folate receptor (FR) to improve encapsulation efficiency, stability, cellular uptake and cytotoxicity against tumor cells. Cell cycle analysis was performed on CAL 27 cell lines, representing FR-positive and FR-negative lines, correspondingly. The thin-layer hydration technique was used to create the nanoliposomes. A comprehensive study of the physicochemical properties of the nanoparticles was performed, including the evaluations of their release rate and effectiveness against tumors.

## Materials and methods

### Materials

In Ludwigshafen, Germany, DSPC (1, 2-Distearoyl-sn-glycero-3-phosphocholine) was purchased from Lipoid GmbH. We purchased the folates DSPE-mPEG3350 and DSPE-PEG (3350) from Avanti Polar Lipids Company in Alabaster, AL. The German company Cell Pharma GmbH was the supplier of Bleomycin hydrochloride. We bought sodium pentanesulfonate, sodium edetate, cholesterol, and MTT (3-(4, 5-dimethylthiazol-2-yl)-2, 5-diphenyltetrazolium bromide) from Sigma-Aldrich Corporation. We bought chloroform and methanol from Merck. We purchased penicillin/streptomycin, fetal bovine serum, RPMI 1640, and DMEM culture media from Invitrogen Corporation. The CAL-27 cell lines were obtained from the National Cell Bank of Iran at the Pasteur Institute of Iran.

### Preparation of nanoliposomes

Nanoliposomes, either unencapsulated or encapsulated with a drug, were prepared using the thin-layer hydration technique. First, specific molar ratios of DSPC/cholesterol/DSPE-mPEG3350 (80:18:6), corresponding to weights of 200 mg, 24 mg and 48 mg, respectively, were prepared in 25 ml of CHCl_3_: MeOH (2:1 v/v) mixture. After that, this combination was put in a flask with a circular bottom, and evaporated under a rotary evaporator at 50°C and 100 rpm. After drying with a nitrogen gas purge, the film was heated in a water bath at 50°C and rehydrated in phosphate buffer saline (PBS, pH 7.2), either with 9.5 mg of the medicine for the test sample or the blank sample without the medication. To ensure targeted distribution, 6.5 mg of FA-PEG3350-DSPE was incorporated into the suspension using the post-insertion technique. Drug-loaded nanoliposomes without FA-PEG3350-DSPE, referred to as L-Bleomycin, were made as a control as well to assess the efficacy of folate-targeted liposomal Bleomycin (FL-Bleomycin). In the process of preparing of L-Bleomycin, The quantity of mPEG3350-DSPE was raised from 48 mg to 53 mg. The mixture was then repeatedly extruded through a polycarbonate syringe filter with a pore size of 200 nm and then 100 nm. Finally, the liposomal Bleomycin was isolated from the free Bleomycin using a Sephadex G-50 column balanced with PBS (pH 7.2).

### Characterization of nanoliposomes

The Zeta-sizer instrument (Nano ZS3600, Malvern Instruments Ltd, Worcestershire, UK) was accustomed to determine the average size and zeta potential of the liposome formulations. After brief sonication at 25°C and 60 W in a bath sonicator, the liposomes were diluted 50-fold in PBS (pH 7.2) and placed in a 1 mL disposable measuring cuvette. The Moluchowski equation, ζ = 12.8 × μe, was used to determine the zeta potential. The electrophoretic mobility is represented by μe and the zeta potential by ζ. A typical polystyrene nanosphere dispersion (DTS5050, Malvern Ltd.) was used to check the accuracy of the device.

Scanning Electron Microscopy (SEM) was also used to characterize the surface morphology and size distribution of synthesized nanoparticles. A diluted sample of nanoparticle was drop-cast onto a silicon wafer, air-dried, and coated with a thin layer of gold using a sputter coater to enhance conductivity. The prepared sample was imaged using a field-emission SEM under high vacuum conditions, with an accelerating voltage of 5–15 kV and a working distance of 8–10 mm. Secondary electron mode was used to capture high-resolution images at magnifications and the obtained images were analyzed using ImageJ software to determine the size and shape of the nanoliposomes, providing insights into their surface features and aggregation behavior.

### Entrapment efficiency (EE %) and loading capacity (LC %)

To evaluate the drug loading and Entrapment efficiency, 1.5 mL of the liposomal formulation was centrifuged in a Beckman ultracentrifuge type 90Ti at 45,000 rpm for 60 min at 4°C. The unencapsulated active ingredient in the supernatant was quantified by HPLC following the methods described in the European Pharmacopoeia 2005. A 25 µL sample was injected into an HPLC system (Knauer, Berlin, Germany) with a PDA 2800 detector and a C18 Nucleosil-100 column (4 mm, 25 cm, 5 µm). Elution began with a mobile phase made up of 10% v/v methanol and 90% v/v of a solution made by dissolving 0.965 g sodium pentanesulfonate in 900 mL acetic acid (4.8 g/L C_2_H_4_O_2_), adding 1.86 g sodium edetate, and diluting to 1,000 mL with the same solvent adjusted to pH 4.3 with ammonia. Following a final 20-min phase at a flow rate of 1.2 mL/min, during which dimethylBleomycin A2 was detected at 254 nm absorbance, the methanol content was raised to 40% v/v over the course of 60 min. The concentration of the medication in the supernatant was measured using a standard curve, and predetermined formulae were utilized to compute the drug loading and Entrapment efficiency:

$$\text{EE}\%=\left(\text{Initial Drug}-\text{Unentrapped Drug}\right)/\text{Initial Drug}\times 100$$

Initial Drug: The quantity of the drug initially incorporated into the formulation, expressed in mg/mL; Unentrapped Drug: The focus of the drug detected in the supernatant, expressed in mg/mL.

$$\text{LC}\%=\left(\text{Entrapped Drug}\right)/\text{Weight of Carrier}\times 100$$

Entrapped Drug: The quantity of drug contained within the liposomes, measured in milligrams (mg); Carrier Weight: The overall weight of the liposomes employed, measured in milligrams (mg).

### *In vitro* drug release study

The effect of the carrier on the drug’s release behavior was evaluated in the drug release study conducted in phosphate buffer saline (PBS) using the membrane diffusion technique. For this purpose, 2 ml of each drug formulation comprising 0.12 mg of the drug along with control samples and free drug were sealed in dialysis bags (cellulose membrane, 12kD cutoff) and immersed in 120 ml of PBS at pH 7.2 and pH 5.2. The experimental setup was kept up at a steady temperature of 37°C and continuously stirred with a magnetic stirrer at 100 rpm. At certain intervals, aliquots were removed from the buffer chamber and replaced with brand new PBS. Prior to analysis, these aliquots were moved through a 0.45 µm syringe filter. High performance liquid chromatography (HPLC) was used to determine the medication concentrations in each aliquot, and a standard curve was used to plot the drug release profiles versus controls.

### *In vitro* cytotoxicity studies

The cytotoxicity of different nanoliposomal Bleomycin formulations on CAL 27 cells was investigated using the MTT assay. These cells were grown in medium containing an additional 10% FBS, 100 mg/mL streptomycin, 100 units/mL penicillin and 0.25 mg/mL amphotericin B and maintained in a 5% CO_2_ humidified atmosphere at 37°C. About 10,000 cells per well were seeded into a 96-well plate containing 200 µL of the medium and allowed to adhere for 24 h. The medium was then substituted with either fresh medium (which served as a negative control) or medium comprising varying concentrations of Bleomycin in nanoliposomal form, and the cells were incubated for a further 48 h. After this time, the medium was substituted with 180 µL fresh medium and 20 µL MTT solution (5 mg/mL in PBS) and incubated for a further 3 h. To stop the reaction, 200 µL of DMSO was added to the medium to dissolve the formazan products. An AccuReader microplate reader (M965 Series, Metertech, and Taipei, Taiwan) was used to record optical density at 570 nm, and GraphPad Prism 6 (GraphPad Software Inc., San Diego, CA) was used to calculate IC50 values.

### Cellular uptake study

Investigating the critical role of cellular absorption of drug-loaded vesicles for their biological effect involved evaluating the efficiency of cellular uptake. To determine this, cells were treated with FL-Bleomycin and L-Bleomycin for 2 hours at four degrees Celsius and thirty-seven degrees Celsius. CAL 27 cells were first cultured at a focus of 3 × 105 cells per well in 24-well plates, either in medium without folate or in medium supplemented with 1 mM folic acid. To release folate bound to the receptors, the cells were quickly washed with cold PBS and saline at pH 3.5 (130 mM NaCl, 20 mM NaOAc) after an overnight incubation. Then, 1 mL of new medium comprising 0.07 mg/mL Bleomycin in different formulations (FL-Bleomycin, L-Bleomycin and ordinary Bleomycin) was added to the old medium and the mixture was incubated at 37°C or 4°C for another 2 hours. After incubation, non-adherent carriers were removed from the cells by washing twice in cold PBS (pH 7.4). After that, the cells were gathered using centrifugation for 5 minutes at 13,000 rpm. 200 μL of 0.5% Triton X-100 was used to lyse the cell pellet after the supernatant was discarded. The lysate was mixed with an equivalent volume of acetonitrile (1:1 v/v) to precipitate the proteins. After further centrifugation at 13,000 rpm for 5 min and filtration through a 0.22 µm filter, 20 µL of the filtrate was analyzed by HPLC to determine the cellular uptake efficiency (CUE%):

CUE%: Amount of Bleomycin in the cells after the incubation time/amount of Bleomycin in the nanoliposomes introduced into the cells × 100.

### Cell cycle analysis

The effects of nanoliposomes on cell cycle progression were looked into using DNA flow cytometry. About 5 × 10^5^ CAL 27 cells per well were first plated in six-well culture plates and cultured at 37°C in a humidified environment with 5% CO_2_ for 24 h. Subsequently, the folate-free medium was substituted with 2 µM of both traditional Bleomycin and FL-Bleomycin, which was done in triplicate. After a 48-h incubation period, the medium was removed and the cells were given trypsin treatment right away, rinsed and harvested. The cells were then fixed in 70% cold ethanol on ice for at least 2 h. They were then centrifuged at 200 *g* for 5 min to form a pellet. This cell pellet was then reinstated in PBS comprising 20 µg/mL propidium iodide (PI), 1 mg/mL RNase and 0.1% Triton X-100 and incubated at 37°C for 15 min in the dark. The DNA content and cell cycle phase distribution were subsequently analyzed using a Partec GmbH flow cytometer from Munster, Germany, equipped with FlowJo software (FlowJo, LLC, and Ashland, OR). Suitable gating strategies were employed to omit cell debris from the examination.

### Western blot

CAL27 cells were seeded in 6-well plates at a density of 4 × 10^5^ cells per well and cultured to 70%–80% confluence, then serum-starved for 24 h to synchronize cell signaling. Subsequently, cells were treated with Folic Acid-Conjugated Nanoliposomes loaded with Bleomycin at designated concentrations, while control groups received either no treatment or Bleomycin alone. After treatment, cells were washed with ice-cold PBS containing 5 mM EDTA and 1 mM sodium orthovanadate, then lysed by scraping into a buffer composed of 1% Triton X-100, 20 mM Tris (pH 8.0), 137 mM NaCl, 10% glycerol, 2 mM EDTA, 1 mM PMSF, 20 µM aprotinin, and 2 mM sodium orthovanadate. The lysates were incubated on ice for 30 min and centrifuged at 14,000 rpm for 15 min at 4°C to remove insoluble material. Protein concentrations were determined using the Bradford assay, and 30 µg of protein per sample were denatured in sample buffer (0.5 M Tris-HCl, pH 6.8; 10% SDS; 1 M DTT; 10% glycerol; 1% bromophenol blue) and boiled. Proteins were resolved on a 7.5% SDS–polyacrylamide gel and transferred onto 0.45-µm nitrocellulose membranes, which were then blocked in 5% non-fat dry milk in TBS-T for 1 h at room temperature. The membranes were subsequently incubated overnight at 4°C with a mouse monoclonal anti–EGF-R antibody (1:1,000 in 5% milk/TBS-T), followed by a 1-h incubation with an HRP-conjugated goat anti-mouse IgG secondary antibody (1:2000 in 5% milk/TBS-T). Protein bands were visualized using an enhanced chemiluminescence detection system, allowing for the assessment of EGF-R expression following treatment with the targeted nanoliposome formulation.

### Stability assessment

Over the course of 45 days, the liposomal nanoparticles’ stability was assessed under physiological settings (37°C, 5% CO_2_). Particle size, zeta potential, Entrapment efficiency, and the polydispersity index (PDI) were measured on a regular basis to evaluate stability.

### Statistical analysis

The data is shown as mean ± standard deviation (SD, n = 3). SPSS software version 19 was utilized for statistical studies, which included the Student's t-test and ANOVA. A significance level of p < 0.05 was set for each test.

## Results

### Characteristics of nanoparticles

Table 1 displays the physicochemical characteristics of nanoliposomal Bleomycin, including the polydispersity index (PDI), mean particle size, zeta potential, entrapment efficiency (EE%), and drug loading (DL%). The data indicates that the particle sizes for all formulations were between 91 and 97 nm, with a PDI not exceeding 0.22, suggesting a uniform and monodisperse distribution of nanoparticles. The zeta potential values confirmed that the nanoparticles have a negative charge, with measurements ranging from −31 to −37 mV. Additionally, the reported entrapment efficiency and drug loading values were around 78% and 11%, respectively.

**TABLE 1:** Physicochemical properties of nanoliposomes.

Formulations	Size (nm)	Zeta potential (mV)	PDI	EE %	DL %
FL-Bleomycin	91 ± 7.4	−37 ± 5.5	0.26 ± 0.2	78 ± 6.5	11 ± 1.5
L-Bleomycin	97 ± 8.3	−31 ± 6.1	0.22 ± 0.11	77 ± 5.5	11 ± 2.5

Each value demonstrates the mean ± SD (n = 3).

**FIGURE 1:**
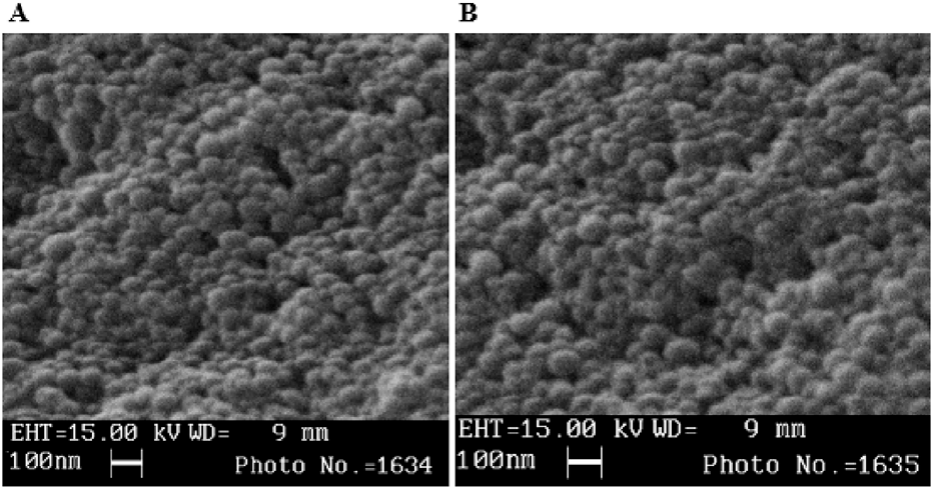
Sem of L-Bleomycin **(A)** and FL-Bleomycin **(B)**.

Based on SEM results (Figure 1), both FL-Bleomycin and L-Bleomycin nanoparticles exhibited sizes below 100 nm, and imaging confirmed that the particles were completely spherical in morphology. This uniform and compact structure is indicative of an optimized formulation potentially favorable for efficient cellular uptake and targeted drug delivery in cancer therapy.

### Drug release study

**FIGURE 2:**
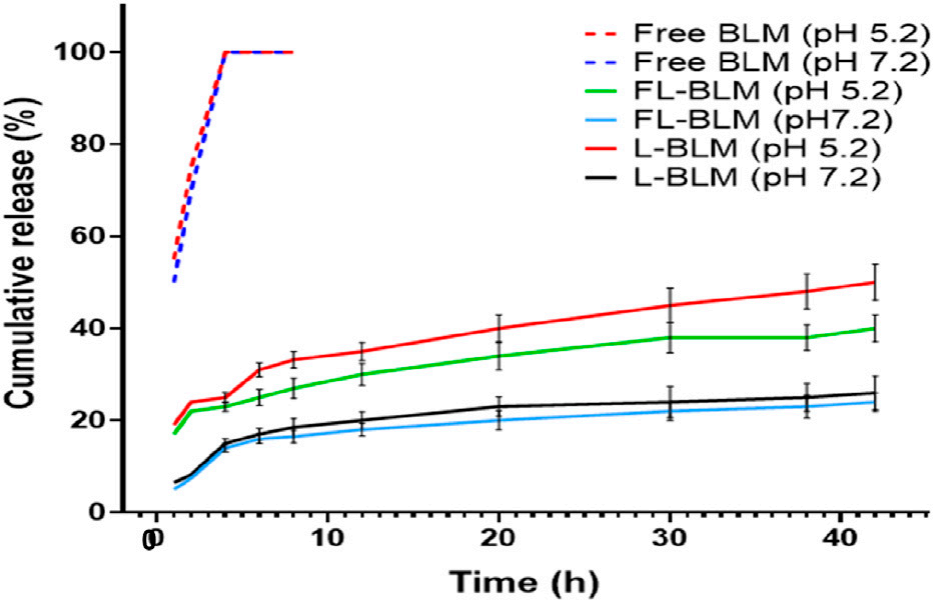
The release profiles of FL-Bleomycin, L-Bleomycin, and free Bleomycin in phosphate buffered saline (PBS) at various pH levels were analyzed using the dialysis method over 42 h at 37°C. The findings are reported as mean ± standard deviation (SD) with a sample size of three (n = 3).

The membrane diffusion method in phosphate buffered saline (PBS) at pH 7.2 and pH 5.2 was used to assess the release of Bleomycin from the FL-Bleomycin and L-Bleomycin formulations. Both formulations showed a consistent release profile without any initial burst release. By the 42-h mark of dialysis in PBS at different pH levels, the release rates for Bleomycin were approximately 24% and 26% at pH 7.2 for FL-Bleomycin and L-Bleomycin, respectively, and about 40% and 50% at pH 5.2, respectively, as shown in Figure 2. This data indicates a notable difference in the release behavior of Bleomycin between the FL-Bleomycin and L-Bleomycin formulations at pH 5.2, although the variations at pH 7.2 were minimal. In contrast, both formulations exhibited significantly slower release rates compared to the unencapsulated Bleomycin formulation at both pH levels, which released nearly completely within 4 h.

### *In vitro* cytotoxicity assay

**FIGURE 3:**
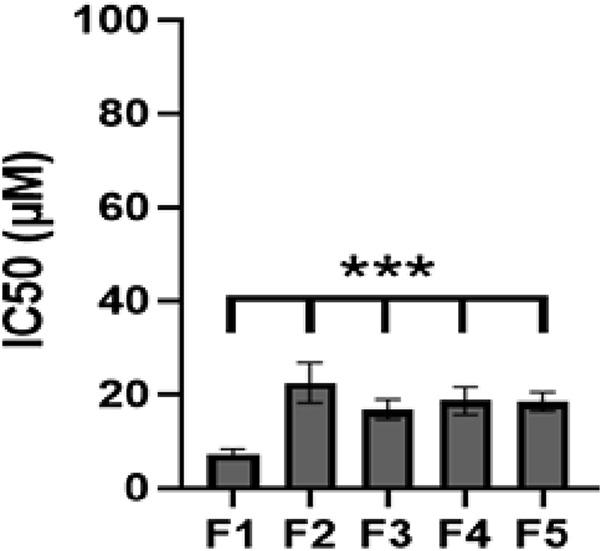
The IC50 values (μM) of various Bleomycin formulations that target various cell lines are presented. Different formulations; F1, FL-Bleomycin in folate-free medium; F2, FL-Bleomycin in standard medium 1 mM folate; F3, FL-Bleomycin in standard medium; F4, L-Bleomycin in standard medium; F5, Conventional Bleomycin in standard medium. (Graph displays mean ± SD; n = 3 independent experiments, p = 0.0002, two-way ANOVA with Šidák test).

After a 48-h incubation at 37°C, the cytotoxicity of Bleomycin -containing nanoliposomes was evaluated against that of conventional Bleomycin at equivalent drug doses on CAL 27, with the results displayed in Figure 3. In both regular and folate-free media, FL-Bleomycin exhibited notably higher cytotoxicity in contrast to conventional Bleomycin in CAL 27 cells, with the most pronounced effect seen in the folate-free medium. This increased cytotoxicity could be mitigated by adding 1 mM folate to the medium.

### Cellular uptake study

**FIGURE 4:**
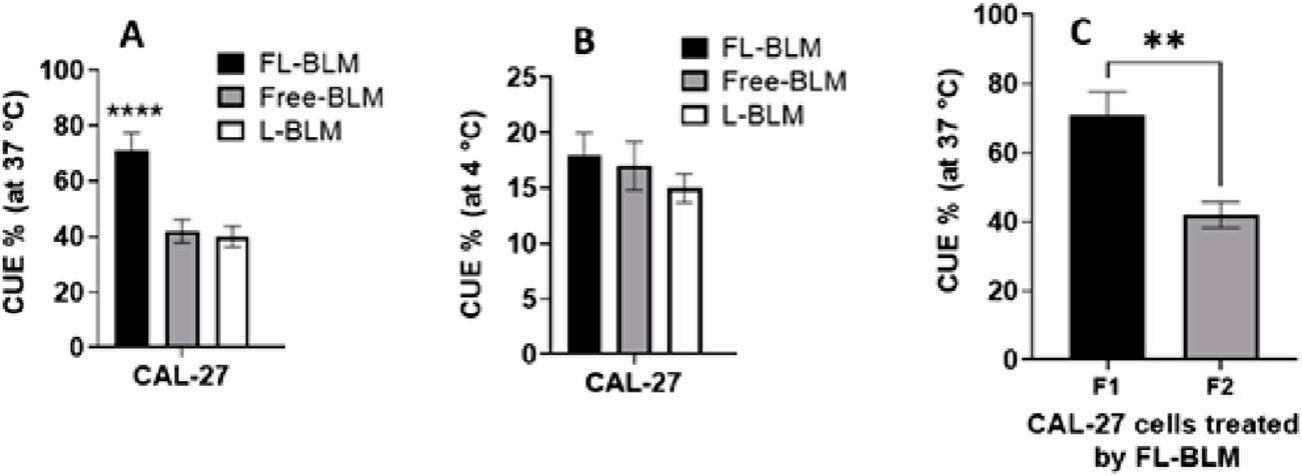
To determine the impact of the folate receptor on the absorption of folate-conjugated nanoliposomes, the efficiency of cellular uptake (CUE %) was assessed **(A)** 37°C, **(B)** 4°C. **(C)** Additionally, the cellular absorption of FL-Bleomycin in CAL 27 cells was examined in relation to the competitive inhibitory impact of free folate, F1, folate-free medium; F2, standard medium 1 mM folate (Graph displays mean ± SD; n = 3 separate tests, p < 0.0001, p = 0.0021, two-way ANOVA with Šidák test).

The role of the folate receptor (FR) in the uptake of folate-conjugated nanoliposomes was explored through a cellular uptake study. As depicted in Figure 4, FL-Bleomycin was significantly more absorbed by CAL 27 cells than by L-Bleomycin and traditional Bleomycin. The efficiency of cellular uptake was found to be time-dependent, with absorption increasing over longer incubation periods (specific data not shown). The inclusion of 1 mM folic acid to the media dramatically decreased the absorption of FL-Bleomycin in CAL 27 cells, underscoring the pivotal role that FR plays in this procedure. At 4°C, the cell lines didn’t show any appreciable differences in cellular absorption between these formulations. This highlights the importance of the folate ligand in enhancing uptake by CAL 27 cells that express the folate receptor.

### Cell cycle analysis

Bleomycin functions as an anticancer agent by inhibiting DNA synthesis, leading primarily to cell cycle arrest in the G2/M phase. To assess the impact of folate-targeted nanoliposomes on cell cycle progression, CAL-27 cells were treated with 2 μM of FL-Bleomycin and conventional Bleomycin for 48 h. The proportion of cells that ceased in the G2/M phase increased statistically significantly when FL-Bleomycin therapy was compared to conventional Bleomycin, according to flow cytometry analysis (Table 2).

**TABLE 2:** Effects of FR-targeted nanoliposomes on the cell cycle progression of CAL-27 cells after a 48-h incubation in folate-free medium.

Formulations	Sub-G1%	G0/G1%	S%	G2/M%
FL- BLEOMYCIN	4.36 ± 1.07	14.42 ± 2.7	47.27 ± 4.73	50.22 ± 5.41
Conventional BLEOMYCIN	14.23 ± 2.45	32.50 ± 3.9	37.19 ± 3.75	24.42 ± 4.10
Nanoliposomes without BLEOMYCIN	12.64 ± 2.0	44.33 ± 4.7	34.29 ± 3.63	15.63 ± 3.02
Negative control (PBS)	9.23 ± 2.1	50.44 ± 5.9	33.44 ± 2.7	12.13 ± 1.8

Each data represents the mean ± SD (n = 3).

### Stability study

The generated liposomal nanoparticles exhibited remarkable stability throughout an extensive 45-day stability assessment conducted under physiological conditions. Particle size, PDI, and zeta potential readings did not show any discernible alterations. Notably, during the investigation, the Entrapment efficiency for each assessed medicinal ingredient consistently exceeded 74% (Table 3).

**TABLE 3:** Stability of liposomal formulations during 45-day storage at 37°C.

Storage (day)	Size (nm)		Zeta potential (mV)		EE %		PDI	
	F1	F2	F1	F2	F1	F2	F1	F2
1	91 ± 7.4	97 ± 8.3	−37 ± 5.5	−31 ± 6.1	78 ± 6.5	77 ± 5.5	0.26 ± 0.20	0.22 ± 0.11
15	91 ± 5.5	99 ± 6.7	−37 ± 1.7	−33 ± 2.9	77 ± 4.4	75 ± 4.7	0.29 ± 0.60	0.27 ± 0.33
30	93 ± 4.8	108 ± 9.7	−34 ± 2.8	−34 ± 2.8	74 ± 5.4	75 ± 5.4	0.29 ± 0.17	0.30 ± 0.20
45	99 ± 6.1	119 ± 7.7	−34 ± 3.1	−36 ± 2.2	75 ± 5.5	74 ± 6.1	0.34 ± 0.11	0.40 ± 0.47

F1, FL-Bleomycin; F2, L-Bleomycin. Each value represents the mean ± SD (n = 3).

### Western blot

**FIGURE 5:**
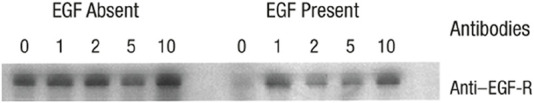
Western blot analysis of EGFR expression in CAL27 cells following treatment with folic acid-conjugated nanoliposomes loaded with bleomycin.

The results obtained from using Acid-Conjugated Nanoliposomes loaded with Bleomycin on CAL27 cells indicate a marked improvement in the targeted delivery of Bleomycin (Figure 5). Specifically, cells treated with these nanoliposomes, compared to control groups, exhibited a dose-dependent decrease in the intensity of the 170-kDa EGF-R band as observed by Western blot analysis. This reduction in expression and/or phosphorylation of EGF-R suggests that the targeted delivery system enhances the efficacy of Bleomycin, thereby potentially reducing side effects related to non-specific drug distribution.

## Discussion

Liposomes are an effective drug delivery system, offering some advantages, including biocompatibility, biodegradability, and low toxicity. Numerous hydrophilic and lipophilic medications can be transported by them to certain target locations. Currently, numerous liposomal formulations are either commercially available or undergoing clinical trials (Chen et al., 2024). Compared to alternative methods, using folate ligands for targeted drug delivery has several benefits. Folic acid, a non-toxic, cost-effective, and non-immunogenic molecule, exhibits a strong affinity for the folate receptor. This receptor is predominantly expressed in various tumors but not in normal cells, making it an ideal target for tumor-specific drug delivery (Chiani et al., 2017b; Frigerio et al., 2019; Scaranti et al., 2020; Rajeshkumar et al., 2024). Several critical factors, such as entrapment or loading efficiencies, particle size and surface charge, release rate, stability, cytotoxicity, and cellular uptake efficiency, must be carefully considered to ensure the effectiveness of a drug delivery system (Chiani et al., 2017b). This study successfully developed and analyzed both non-folate (L-Bleomycin) and folate-targeted (FL-Bleomycin) nanoliposomes loaded with Bleomycin. These nanoliposomes exhibited particle sizes ranging from 91 to 97 nm and zeta potentials from −31 to −37 mV. These dimensions are ideal for drug delivery systems, as particles within the 10–100 nm range can evade the reticuloendothelial system, avoid renal excretion, and effectively penetrate tumor cells. The zeta potential, or surface charge, is crucial for maintaining the stability of these colloidal dispersions. A zeta potential greater than 30 mV typically ensures stability through electrical repulsion among particles (Müller et al., 2001). The produced nanoliposomes’ zeta potential values were mostly responsible for their high enough zeta potential to sustain their suspension stability during 2 months of storage at 37°C.

## Discussion and analysis of SEM results

The Scanning Electron Microscopy (SEM) results provide critical insights into the morphological and size characteristics of the folic acid-conjugated nanoliposomes (FL-Bleomycin) and conventional nanoliposomes (L-Bleomycin) loaded with Bleomycin. Both formulations exhibited particle sizes below 100 nm, with imaging confirming a completely spherical morphology. This uniform and compact structure is a hallmark of an optimized nanoparticle formulation, which is highly desirable for efficient cellular uptake and targeted drug delivery in cancer therapy (Zare-Zardini et al., 2020). The sub-100 nm size range is particularly advantageous, as it falls within the optimal size window for enhanced permeability and retention (EPR) effect in tumor tissues. This size facilitates deeper penetration into tumor sites while minimizing rapid clearance by the reticuloendothelial system (RES) (Alemi et al., 2018). Additionally, the spherical morphology observed in both formulations suggests high stability and consistency in particle formation, which is essential for reproducible drug delivery and therapeutic outcomes (Kumbham et al., 2023; Salehan et al., 2025). The presence of folic acid conjugation in the FL-Bleomycin nanoparticles is expected to further enhance their targeting capability by leveraging the overexpression of folate receptors on cancer cells, such as CAL27. This targeted approach not only improves drug delivery efficiency but also reduces off-target effects, thereby increasing the overall safety and efficacy of the treatment (Salehan et al., 2025).

Another crucial factor influencing the therapeutic efficacy of the encapsulated drug is the drug entrapment or loading efficiency. We achieved an entrapment efficiency of approximately 78%, significantly higher than previously reported values (Koshkaryev et al., 2013; Firth et al., 1988). These results suggest the appropriate selection and quantities of materials for nanoliposome preparation. *In vitro* cytotoxicity assays revealed that folate-targeted (FL-Bleomycin) nanoliposomes were notably more cytotoxic than non-folate (L-Bleomycin) and conventional drugs in CAL-27 cells. This increased toxicity, indicated by a lower IC50, is attributed to differences in cell type and formulation specifics. CAL-27 cells express folate receptors (FR). The selection of CAL-27 cells as a model for folate receptor (FR)-targeted therapy is grounded in established evidence of FR overexpression in oral squamous cell carcinoma (OSCC) and validated experimental observations. FR, particularly the alpha isoform (FRα), is widely documented to be upregulated in OSCC clinical samples and cell lines, serving as a promising target for ligand-directed therapies (Bhattacharya et al., 2023; Patrojanasophon et al., 2025). The inclusion of folic acid as a targeting ligand in the nanoliposome formulation significantly enhanced cellular uptake and thus the cytotoxicity of Bleomycin in FR-positive cells. Previous studies have established the importance of FR in improving the effectiveness of conjugates that target folate (Chiani et al., 2017b; Hilgenbrink and Low, 2005; Yoo and Park, 2004; Wu et al., 2006). Conventional Bleomycin struggles to penetrate cells, but when encapsulated within folate-targeted nanoliposomes, it efficiently enters cells via endocytosis. The controlled and prolonged release of Bleomycin from these nanoliposomes likely contributes to the enhanced cytotoxicity observed with FL-Bleomycin in contrast to conventional Bleomycin, as this sustained release increases cellular uptake and protects the drug from adverse environmental factors. Additionally, cell cycle research demonstrated that FL-Bleomycin, unlike the standard formulation, dramatically enhanced G2/M phase cell cycle arrest in CAL-27 cells. Similar findings by Alomrani et al. (Alomrani et al., 2011) demonstrated that lipid flexibility fulfills a crucial function in determining the cytotoxicity and cellular death pathways of nanoliposomes. Subsequent investigations have shown the efficacy of folate-targeted liposomes in enhancing anticancer activity across various cancer cell types (Sriraman et al., 2016).

The Western blot analysis of CAL27 cells treated with folic acid-conjugated nanoliposomes loaded with Bleomycin provides compelling evidence of the enhanced targeted delivery and therapeutic efficacy of our formulation. The results demonstrate a dose-dependent decrease in the intensity of the 170-kDa EGF-R band compared to control groups. This finding is significant, as the epidermal growth factor receptor (EGF-R) is a critical marker in cancer progression, particularly in oral squamous cell carcinomas like CAL27 (Aamir et al., 2024; Chen et al., 2003). The observed reduction in EGF-R expression and/or phosphorylation suggests that the folic acid-conjugated nanoliposomes effectively deliver Bleomycin to the cancer cells, leading to enhanced therapeutic activity. This targeted approach minimizes off-target effects, which are often associated with conventional chemotherapy. By specifically engaging folate receptors on the cancer cell surface, the nanoliposomes ensure that Bleomycin is internalized more efficiently, thereby increasing its cytotoxic effect on cancer cells while potentially sparing healthy tissues (Loureiro et al., 2015; Mirzaghavami et al., 2022). Furthermore, the dose-dependent nature of the response underscores the specificity and controllability of our delivery system (Bittleman et al., 2018; Ebrahimnejad et al., 2022). This is particularly important for Bleomycin, a potent chemotherapeutic agent known for its dose-limiting toxicity. The ability to achieve therapeutic efficacy at lower doses could significantly reduce adverse effects, improving patient outcomes and treatment compliance.

## Conclusion

We successfully created an FR-targeted nanoliposomal formulation of Bleomycin, which greatly improved antitumor activity and cellular uptake in oral cancer cells. This new formulation was more effective at inducing G2/M phase cell cycle arrest in contrast to conventional Bleomycin. All things considered; our discoveries indicate that focused on medicine administration of Bleomycin using FR might greatly better the effectiveness of cancer treatments.

## Data Availability

The datasets presented in this article are not readily available because nond. Requests to access the datasets should be directed to hzare@meybod.ac.ir.
